# Transitioning from Lupus Low Disease Activity State to remission in systemic lupus erythematosus: real-world evidence

**DOI:** 10.3389/fimmu.2025.1546306

**Published:** 2025-03-20

**Authors:** Dai Gao, Lanlan Ji, Xiaohui Zhang, Yanjie Hao, Wenhui Xie, Yong Fan, Zhuoli Zhang

**Affiliations:** ^1^ Department of Rheumatology and Clinical Immunology, Peking University First Hospital, Beijing, China; ^2^ National Clinical Research Center for Skin and Immune Diseases, Peking University First Hospital, Beijing, China

**Keywords:** systemic lupus erythematosus, lupus low disease activity state, remission, prednisone dose, cohort study

## Abstract

**Objectives:**

To identify predictors and barriers to achieving remission in systemic lupus erythematosus (SLE) patients after attaining Lupus Low Disease Activity State (LLDAS).

**Methods:**

This study included patients from the Sle to TARget (STAR) cohort who did not fulfill LLDAS at baseline. The Kaplan-Meier method was used to estimate the cumulative probabilities of remission or flare after LLDAS attainment. Univariate and multivariable Cox proportional hazards models were employed to identify predictors of time to remission. Barriers impeding remission achievement were also investigated.

**Results:**

Of 586 enrolled patients, 480 achieved LLDAS within 20.4 months (IQR 13.4-37.1). Among these, 369 patients who did not achieve remission simultaneously with LLDAS attainment and had ongoing follow-up were included in further analysis. Subsequently, 297 (80.5%) patients achieved remission, with median times to remission and flare of 12.4 and 24.4 months, respectively. Independent predictors of a shorter time to remission included older age at disease onset (HR 1.012, 95%CI=1.004-1.020, *P*=0.002), arthritis (HR 1.481, 95%CI=1.113-1.969, *P*=0.007), and gastrointestinal involvement (HR 1.994, 95%CI=1.230-3.232, *P*=0.005). Conversely, anemia (HR 0.564, 95%CI=0.428-0.743, *P*<0.001) was a risk predictor. Higher disease activity defined by SLE Disease Activity Index 2000 (HR 0.691, 95%CI=0.632-0.757, *P*<0.001) or the Physician’s Global Assessment (HR 0.062, 95%CI=0.031-0.127, *P*<0.001) and the presence of rash (HR 0.156, 95%CI=0.049-0.499, *P*=0.002), anti-dsDNA positivity (HR 0.513, 95%CI=0.403-0.654, *P*<0.001), hypocomplementemia (HR 0.468, 95%CI=0.346-0.632, *P*<0.001), or thrombocytopenia (HR 0.138, 95%CI=0.051-0.377, *P*<0.001) at the time of LLDAS attainment also demonstrated negative associations with remission. Patients maintaining hydroxychloroquine (HR 1.662, 95%CI=1.115-2.477, *P*=0.013) or cyclophosphamide (HR 3.468, 95%CI=1.959-6.141, *P*<0.001) regimens at LLDAS exhibited a shorter time to remission. Moreover, 68.7% of patients failed to achieve remission at the visit preceding remission solely due to prednisone doses of ≥5 mg/day, while other criteria impeded only 5.7-8.4% of cases.

**Conclusions:**

Achieving rapid remission after LLDAS attainment remains challenging for most SLE patients, mainly due to difficulties in reducing prednisone dosage to ≤5 mg/day.

## Introduction

Systemic lupus erythematosus (SLE) is a chronic, complex autoimmune disorder characterized by a diverse spectrum of clinical manifestations. Although survival rates among patients with SLE have improved in recent decades owing to advances in early diagnosis and pharmacotherapy, the standardized mortality ratio for SLE remains 2.87 times as high as that of the general population (1–4).

The treat-to-target (T2T) paradigm in systemic lupus erythematosus (SLE) has evolved substantially since its introduction in 2014. Central to this paradigm are the Lupus Low Disease Activity State (LLDAS) and the Definition of Remission in SLE (DORIS), both of which have been validated as key therapeutic endpoints (5, 6). While DORIS criteria represent a more stringent therapeutic target and some studies suggest its potential benefits on clinical outcomes (7, 8), evidence from both randomized controlled trials and real-world cohorts consistently demonstrates substantially lower attainment rates compared to LLDAS. In an analysis of the multinational Asia Pacific Lupus Collaboration (APLC) cohort comprising 3,811 patients, 52.2% reached LLDAS for at least 50% of the time observed, compared with only 36.8% achieving DORIS-defined remission (9). Similarly, longitudinal data from the Hopkins Lupus Cohort showed that patients spent 50% of the follow-up period in LLDAS, but only 27% in DORIS remission (7). Our previous study further revealed that treatment-naïve SLE patients required an average of 2.6 years to achieve remission, whereas LLDAS was reached in just 1.4 years (10). Moreover, a pooled analysis of five phase III trials involving 1,869 SLE patients treated with belimumab indicated that merely 17% attained LLDAS at week 52, with only 8% achieving DORIS remission (11).

Given the consistently lower attainment rates of DORIS remission compared to LLDAS, this study aims to investigate the factors associated with and barriers to achieving remission in SLE patients who have attained LLDAS. Understanding these determinants may help optimize therapeutic strategies for patients with SLE.

## Materials and methods

### Patients

This study included patients from the Sle to TARget (STAR) cohort, previously known as the Peking University First Hospital SLE cohort, which is a single-center, prospective observational cohort established in 2007. The design of this cohort has been previously detailed (10, 12, 13). In this study, we included patients from the STAR cohort who were enrolled up until December 2019. All patients fulfilled either the 1997 revised SLE American College of Rheumatology (ACR) classification criteria or the 2012 Systemic Lupus International Collaborating Clinics (SLICC) classification criteria, depending on their enrolment dates. The ethics committee of Peking University First Hospital granted approval for this cohort, and all patients provided informed consent at enrolment.

This study constituted a retrospective re-examination of data from the STAR cohort. The inclusion criteria for this analysis were defined for patients in the STAR cohort who met the following conditions: 1) did not comply with the LLDAS criteria at baseline; 2) had a minimum follow-up period of 12 months; and 3) possessed adequate data to evaluate target achievement and flare occurrence at baseline and each follow-up visit.

### Data collection

The gender, age at disease onset, and complications such as antiphospholipid syndrome (APS) and Sjogren’s syndrome (SS) were documented for each patient. The age at disease onset was identified as the age when the patient first exhibited symptoms or laboratory abnormalities related to SLE. Complications were diagnosed according to the 2006 Sydney criteria for APS and the 2016 ACR/EULAR criteria for SS (14–16).

Follow-up data were collected from cohort entry to December 2023. A lack of follow-up data for twelve consecutive months was considered as loss. For the baseline and each follow-up visit, clinical manifestations, laboratory abnormalities, and medications were documented. A rheumatologist assessed the disease activity of SLE using the SLE Disease Activity Index 2000 (SLEDAI-2K) and the Physician’s Global Assessment (PGA) (range 0–3.0) at the baseline and each subsequent visit (17, 18). Flare, as defined by the SLE Flare Index, was evaluated at each visit (19, 20). Organ damage was assessed using the SLE SLICC/ACR Damage Index (SDI) (21).

To further enhance the reliability of the assessment, any instance of a patient experiencing a continuous absence of medical records exceeding 12 months in this study was classified as loss to follow-up, even if the patient had subsequent follow-up records. In cases of missing data, we employed a last observation carried forward (LOCF) approach, utilizing data from the previous follow-up.

### Definitions of LLDAS and remission

At baseline and each visit, all the patients were evaluated for LLDAS or remission attainment. The definitions of LLDAS included: (a) SLEDAI-2K ≤ 4, with no activity in major organ systems (renal, central nervous system, cardiopulmonary, vasculitis, fever), and no hemolytic anemia or gastrointestinal active; (b) No new features of lupus disease activity compared with the previous assessment; (c) PGA ≤ 1; (d) Prednisone (or equivalent) dose ≤7.5 mg/day; and (e) Well-tolerated standard maintenance doses of Ims (22). The definitions of remission included: (a) clinical SLEDAI-2K = 0 (irrespective of serology); (b) PGA <0.5; (c) Prednisone (or equivalent) dose ≤5 mg/day; (d) Stable Immunosuppressants including biologics (23, 24).

### Statistical analysis

Descriptive statistics were presented as the median (interquartile range [IQR, 25th percentile-75th percentile]) for continuous variables and as numbers (frequencies or percentages) for categorical variables. Comparisons between continuous variables were conducted using the Mann-Whitney U test, while Fisher’s exact test was employed for categorical variables. The Kaplan-Meier method was used to estimate the cumulative probabilities of remission or flare following LLDAS attainment. Univariate and multivariable Cox proportional hazards models were utilized to identify predictors of time to remission. Then, variables with a p-value ≤ 0.2 in the univariate analysis were included in the multivariable model, and those that remained significant were retained. Additionally, a Venn diagram was used to illustrate the fulfilment of each remission criterion at different visits. All statistical analyses were performed using STATA version 16.0 (StataCorp, College Station, TX, USA) for Windows, with a p-value of <0.05 considered statistically significant.

## Results

### Patients

**Figure 1:**
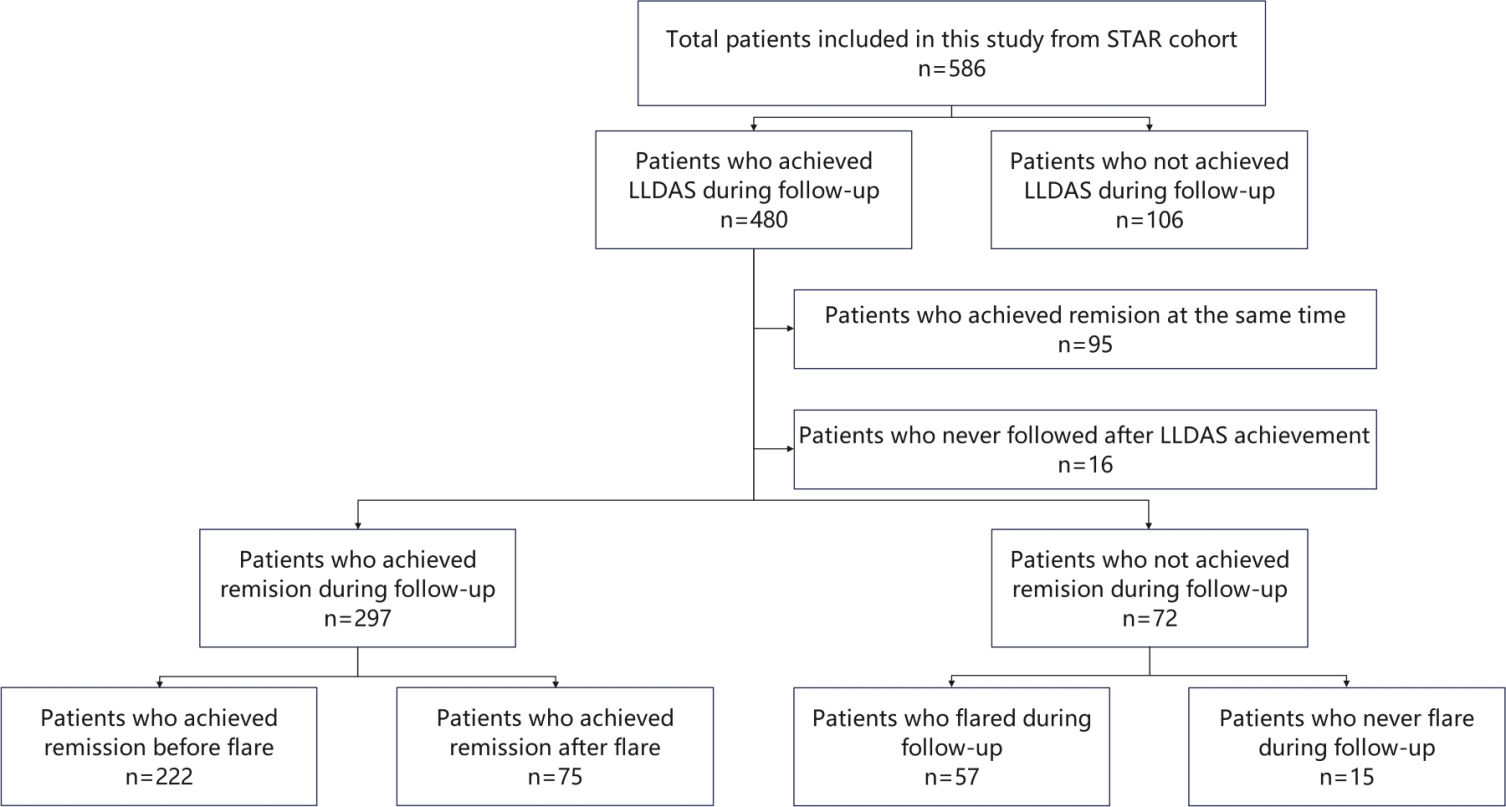
Study design. STAR, treat Systemic lupus erythematosus to TARget; LLDAS, Lupus Low Disease Activity State.

In this study, 586 patients from the STAR cohort were included. During the median 61.9 (40.7-93.1) months of follow-up, total 480 patients achieved LLDAS at least once and were included in the subsequent analysis (**Figure 1**). At baseline, the median SLEDAI, cSLEDAI and PGA scores were 8 (5–16), 2 (6-12) and 1.6 (1.0-1.9), while the median daily prednisone (or equivalent) dose was 50 (30-60) mg/day. The comorbidities, clinical manifestations, and laboratory disorders of these SLE patients were shown in **Table 1**. The median time to LLDAS was 20.4 (13.4-37.1) months. Meanwhile, the demographics, disease activity, previous organ damage, and medications at the time of LLDAS attainment were detailed in **Table 2**.

**Table 1:** Demographic and clinical characteristics of 480 SLE patients who attained LLDAS during follow-up.

Characteristics	Median (IQR) or number (%)
Female	425 (88.5)
Age at disease onset, years	29.8 (22.9-41.3)
Comorbidities	
SS	73 (15.2)
APS	17 (3.5)
Clinical manifestations ever	
Fever	99 (20.6)
Mucosal ulcers	84 (17.5)
Alopecia	204 (42.5)
Rash	266 (55.4)
Raynaud’s phenomenon	85 (17.7)
Arthritis	99 (20.6)
Myositis	20 (4.2)
Serositis	98 (20.4)
Neuropsychiatric manifestations	61 (12.7)
Nephritis	266 (55.4)
Hemolytic anemia	28 (5.8)
Gastrointestinal involvement	28 (5.8)
Laboratory disorders ever	
ANA	476 (99.2)
Anti-Sm	110 (22.9)
Anti-nRNP	203 (42.3)
Anti-rRNP	150 (31.3)
Anti-SSA	278 (57.9)
Anti-SSB	76 (15.8)
Anti-dsDNA	414 (86.3)
Hypocomplementemia	400 (83.3)
Anemia	357 (74.4)
Leukopenia	192 (40.0)
Thrombocytopenia	122 (25.4)
Elevated serum creatinine	40 (8.3)

LLDAS, Lupus Low Disease Activity State; IQR, inter quartile range; SS, Sjogren’s syndrome; APS, antiphospholipid syndrome; ANA, antinuclear antibody.

**Table 2:** Demographic and clinical characteristics of 480 SLE patients at the time of LLDAS attainment.

Characteristics	At the visit of LLDAS attainment
Age, years	35.4 (28.2-49.1)
Disease duration, years	3.8 (1.8-7.9)
PGA	0.3 (0.2-0.4)
SLEDAI-2K*	2 (0-2)
Alopecia	5 (1.0)
Rash	7 (1.5)
Anti-dsDNA positive	227 (47.3)
Hypocomplementemia	101 (21.0)
Leukopenia	8 (1.7)
Thrombocytopenia	11 (2.3)
SDI	1 (0-2)
Treatments	
Prednisone (or equivalent) dose at LLDAS, mg/day	7.5 (6.3-7.5)
Hydroxychloroquine	430 (89.6)
Immunosuppressants	318 (66.2)
Mycophenolate mofetil	130 (27.1)
Azathioprine	79 (16.5)
Methotrexate	48 (10.0)
Leflunomide	30 (6.3)
Cyclosporine A	21 (4.4)
Cyclophosphamide	20 (4.2)
Tacrolimus	4 (0.8)

*Items of SLEDAI-2K not shown were all negative. LLDAS, Lupus Low Disease Activity State; PGA, physician’s global assessment; SLEDAI-2K, systemic lupus erythematosus disease activity index 2000; SDI, Systemic Lupus International Collaborating Clinics/American College of Rheumatology Damage Index.

### Remission and flare after LLDAS

A total of 369 out of 480 patients who did not achieve remission simultaneously with LLDAS attainment and had ongoing follow-up were included in further analysis (**Figure 1**). The median follow-up duration after LLDAS attainment for these patients was 51.5 (32.2-75.4) months.

Among these SLE patients, 80.5% (297/369) achieved remission during subsequent follow-up, and 74.7% (222/297) of them did not experience a flare before achieving remission. The median (IQR) time to remission was 12.4 (6.3-32.9) months, with a 95% confidence interval of 10.7 to 14.0 months. The cumulative probabilities of achieving remission after LLDAS attainment at months 6, 12, 24, 36, 48, and 60 were 36.3%, 59.0%, 75.3%, 81.4%, 85.7%, and 89.4%, respectively.

A total of 65.5% (242/369) of patients experienced at least one flare after LLDAS attainment during the entire follow-up period, with up to 54.5% (132/242) of the first flares occurring before remission. The median (IQR) time to flare was 24.4 (8.8-80.0) months, with a 95% confidence interval of 20.3 to 29.8 months. The cumulative probabilities of flare after LLDAS attainment at months 6, 12, 24, 36, 48, and 60 were 16.6%, 30.4%, 48.9%, 63.3%, 71.4%, and 73.8%, respectively.

**Supplementary Table 1** presents the demographic and clinical characteristics of patients who achieved remission or experienced a flare earlier after LLDAS attainment. We found that patients who achieved remission before experiencing a flare during subsequent follow-ups were older and had lower rates of rash, hemolytic anemia, leukopenia, and thrombocytopenia, but a higher rate of gastrointestinal involvement. Conversely, at the time of LLDAS attainment, patients who experienced a flare before achieving remission had higher PGA and SLEDAI-2K scores, larger proportions of rash, anti-dsDNA positivity, and hypocomplementemia, and a lower proportion of cyclophosphamide usage.

### Predictors of remission

The factors associated with the time to remission after LLDAS attainment were identified through univariable (**Supplementary Table 2**) and multivariable (**Table 3**) Cox regression analyses.

**Table 3:** Factors associated with time to remission after LLDAS attainment in 369 patients based on multivariable Cox model.

Characteristics	Model 1		Model 2		Model 3	
	HR (95% CI)	*P*	HR (95% CI)	*P*	HR (95% CI)	*P*
Age at disease onset, years	1.012 (1.004-1.020)	0.002	1.009 (1.002-1.017)	0.019	1.012 (1.004-1.019)	0.003
Arthritis ever	1.481 (1.113-1.969)	0.007	1.504 (1.139-1.987)	0.004	1.489 (1.129-1.966)	0.005
Gastrointestinal involvement ever	1.994 (1.230-3.232)	0.005	2.013 (1.252-3.239)	0.004	1.858 (1.157-2.983)	0.010
Anemia ever	0.564 (0.428-0.743)	<0.001	0.593 (0.452-0.778)	<0.001	0.628 (0.479-0.823)	0.001
Rash at LLDAS	0.156 (0.049-0.499)	0.002				
Anti-dsDNA positive at LLDAS	0.513 (0.403-0.654)	<0.001				
Hypocomplementemia at LLDAS	0.468 (0.346-0.632)	<0.001				
Thrombocytopenia at LLDAS	0.138 (0.051-0.377)	<0.001				
SLEDAI-2K at LLDAS			0.691 (0.632-0.757)	<0.001		
PGA at LLDAS					0.062 (0.031-0.127)	<0.001
Prednisone (or equivalent) dose at LLDAS, mg/day			0.922 (0.804-1.056)	0.241	0.889 (0.776-1.017)	0.086
Hydroxychloroquine at LLDAS	1.662 (1.115-2.477)	0.013	1.631 (1.085-2.452)	0.019	1.725 (1.136-2.618)	0.011
Cyclophosphamide at LLDAS	3.468 (1.959-6.141)	<0.001	3.500 (1.995-6.141)	<0.001	4.308 (2.422-7.663)	<0.001

In Model 1, the components of LLDAS (daily prednisone dose, SLEDAI-2K, and PGA) were not included. In subsequent models, either SLEDAI-2K (Model 2) or PGA (Model 3) was added to the respective model along with the daily prednisone dose for adjustment. To avoid collinearity, SLEDAI-2K and PGA were not included in the same statistical model, and the individual components of SLEDAI were also excluded. LLDAS, Lupus Low Disease Activity State; HR, hazard ratio; CI, confidence interval; SLEDAI-2K, Systemic Lupus Erythematosus Disease Activity Index 2000; PGA, physician’s global assessment.

We found that patients with an older age at disease onset (HR 1.012, 95%CI=1.004-1.020, *P*=0.002), or those who had ever manifested arthritis (HR 1.481, 95%CI=1.113-1.969, *P*=0.007) or gastrointestinal involvement (HR 1.994, 95%CI=1.230-3.232, *P*=0.005) had a shorter time to remission after LLDAS attainment. Conversely, patients who had ever manifested anemia (HR 0.564, 95%CI=0.428-0.743, *P*<0.001) had a longer time to remission. At the time of LLDAS attainment, higher SLEDAI-2K (HR 0.691, 95%CI=0.632-0.757, *P*<0.001) or PGA (HR 0.062, 95%CI=0.031-0.127, *P*<0.001) scores, as well as the presence of rash (HR 0.156, 95%CI=0.049-0.499, *P*=0.002), anti-dsDNA positivity (HR 0.513, 95%CI=0.403-0.654, *P*<0.001), hypocomplementemia (HR 0.468, 95%CI=0.346-0.632, *P*<0.001), or thrombocytopenia (HR 0.138, 95%CI=0.051-0.377, *P*<0.001), were identified as negative factors for achieving remission. Additionally, patients who continued hydroxychloroquine (HR 1.662, 95%CI=1.115-2.477, *P*=0.013) or cyclophosphamide (HR 3.468, 95%CI=1.959-6.141, *P*<0.001) treatment at the time of LLDAS attainment had a shorter time to remission.

**Figure 2:**
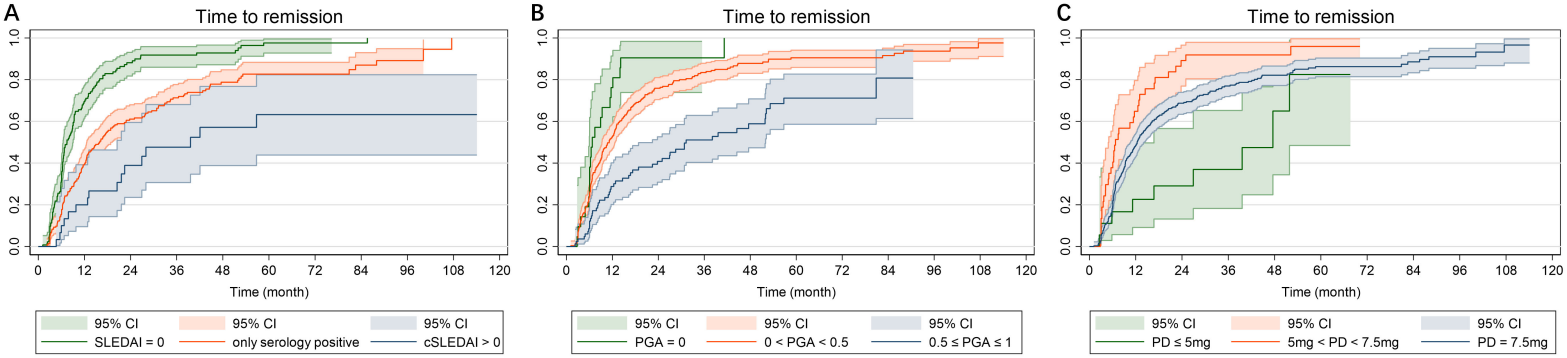
Kaplan-Meier curves in 369 SLE patients who continuous to be followed after LLDAS attainment. **(A)** Time to remission grouped by SLEDAI-2K at LLDAS attainment; **(B)** Time to remission grouped by PGA at LLDAS attainment; **(C)** Time to remission grouped by PD at LLDAS attainment. SLE, systemic lupus erythematosus; LLDAS, Lupus Low Disease Activity State; PD, prednisone dose.

The Kaplan-Meier curves illustrating the time to remission, grouped by disease activity or prednisone dose at LLDAS attainment, are presented in **Figure 2**.

### Barriers of remission

The barriers to remission achievement were also examined. The median (IQR) time delay for the 297 patients who achieved remission following LLDAS attainment was 9.3 (6.0-17.3) months.

**Figure 3:**
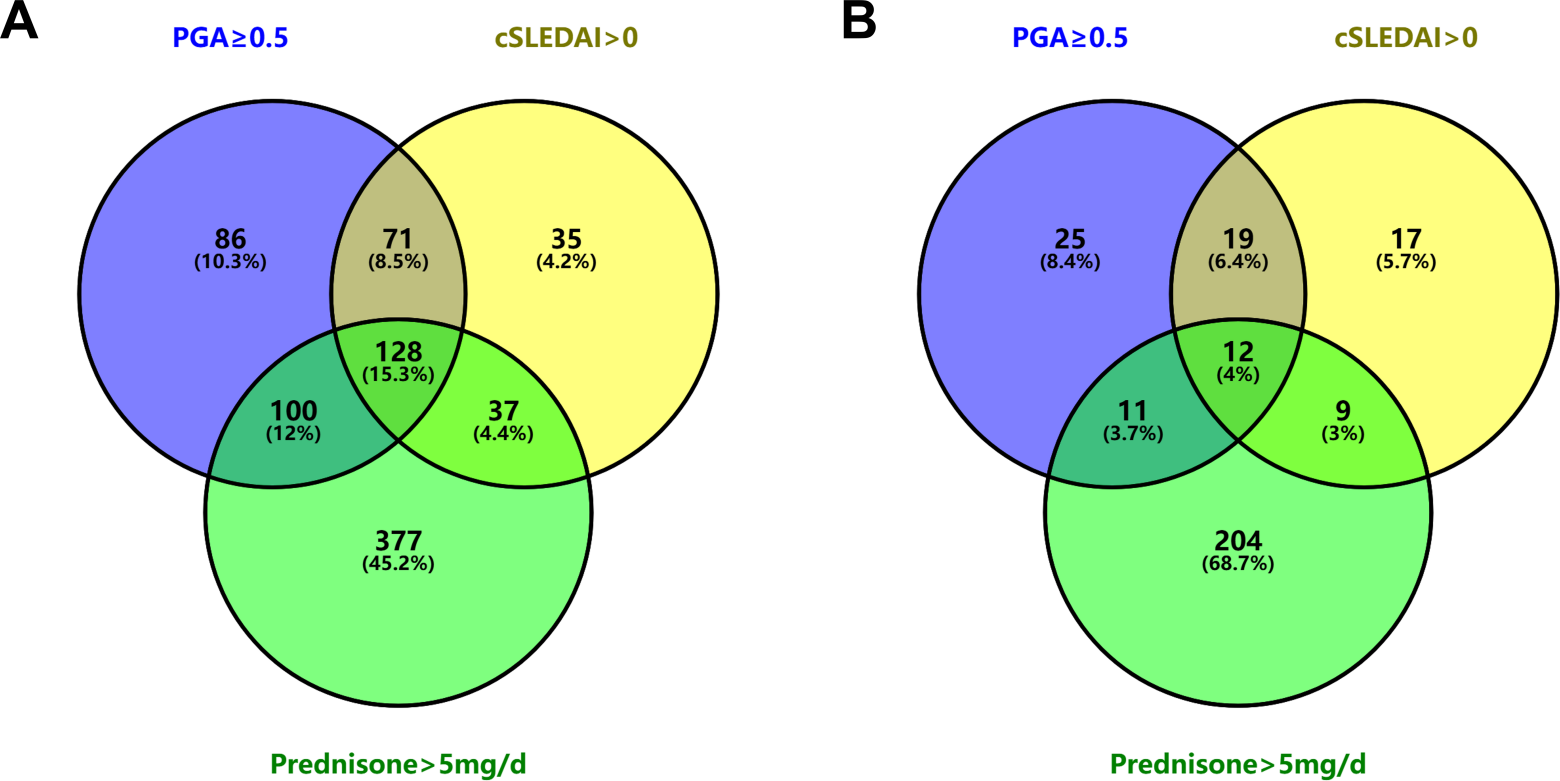
Venn diagram illustrating the unfulfillment of each remission criterion. **(A)** At 834 visits between LLDAS attainment and remission achievement. **(B)** At the visits immediately preceding remission achievement in 297 SLE patients. LLDAS, Lupus Low Disease Activity State; SLE, systemic lupus erythematosus.

During this delay period, 834 visits were conducted. Of these visits, 46.2%, 34.4%, and 77.0% did not meet the criteria of PGA < 0.5, cSLEDAI = 0, or prednisone dose ≤ 5 mg/day, respectively. In contrast, at the visit immediately preceding remission achievement for each patient (median (IQR) time interval of 3.7 (3.0-6.3) months), the rates were 22.6%, 19.2%, and 79.5%, respectively (as shown in **Table 4**). The combinations that satisfied different criteria for remission at various visits are depicted by Venn diagram in **Figure 3**.

**Table 4:** Clinical characteristics of 297 patients at the visit before remission achievement.

Characteristics	All visits before remission after LLDAS attainment (n=834)*	Visit immediately preceding remission achievement of each patient (n=297)*
PGA	0.4 (0.3-0.6)	0.3 (0.2-0.4)
PGA≥0.5	385 (46.2)	67 (22.6)
cSLEDAI	0 (0-1)	0 (0-0)
cSLEDAI>0	287 (34.4)	57 (19.2)
Fever	8 (1.0)	1 (0.3)
Mucosal ulcers	9 (1.1)	2 (0.7)
Alopecia	31 (3.7)	8 (2.7)
Rash	58 (7.0)	11 (3.7)
Arthritis	12 (1.4)	1 (0.3)
Myositis	1 (0.1)	0
Serositis	3 (0.4)	0
Neuropsychiatric manifestations	4 (0.5) **	0
Nephritis	91 (10.9)	15 (5.1)
Proteinuria	34 (4.1)	4 (1.3)
Hematuria	50 (6.0)	7 (2.4)
Pyuria	36 (4.3)	5 (1.7)
Urinary casts	6 (0.7)	0
Leukopenia	55 (6.6)	13 (4.4)
Thrombocytopenia	45 (5.4)	9 (3.0)
Prednisone (or equivalent) dose, mg/day	7.5 (6.3-7.5)	6.3 (6.3-7.5)
Prednisone (or equivalent) dose >5mg/day	642 (77.0)	236 (79.5)

*Median (IQR) or number (%). ** 1 seizure, 1 visual disturbance and 2 cranial nerve disorder.

## Discussion

Our study examined the probability of remission after LLDAS attainment in SLE patients from the STAR cohort. For consistency, we excluded patients who met LLDAS criteria upon cohort enrolment.

Firstly, our study has demonstrated that achieving rapid remission after LLDAS attainment is challenging for the majority of patients. Real-world evidence indicates that remission is less attainable than LLDAS due to more stringent criteria (7–10, 25, 26). Studies conducted on the Asian-Pacific Lupus Collaboration cohort and Hopkins Lupus Cohort have reported that the percentage of patients in remission for at least half or the entire follow-up period was approximately one-third to half lower than that for LLDAS (7, 9). Our findings indicate that only approximately one-fifth of the patients achieved remission simultaneously with LLDAS attainment. For patients who continued to be monitored after attaining LLDAS, the median time to remission was 9.3 (6.0-17.3) months. Approximately two-thirds of these patients experienced disease flare after LLDAS attainment during follow-up, with more than half of these flares occurring before achieving remission. These results collectively suggest that achieving remission remains a significant challenge, even for patients who have attained LLDAS.

Secondly, this study sought to determine the impact of disease activity at the time of LLDAS attainment on subsequent disease progression trends. Previous studies have demonstrated that patients with lower disease activity, as assessed by SLEDAI or PGA, have a higher probability of attaining or maintaining remission (26, 27). Additionally, Wilhelm et al. reported that low C3 levels and hematological activity at baseline were associated with a prolonged time to remission (27). The present study corroborated these findings and further identified the presence of rash, thrombocytopenia, positive anti-dsDNA antibodies, or hypocomplementemia at LLDAS attainment as risk factors impeding the achievement of remission.

Thirdly, the present study identified that the primary barrier to achieving remission in SLE patients is the failure to reduce prednisone dose to ≤5 mg/day. In a previous study of treatment-naïve SLE patients, we observed that only 78.4% achieved a prednisone dose ≤5 mg/day during follow-up, in contrast to 96.3% and 91.3% who reached cSLEDAI-2K scores of 0 or PGA <0.5, respectively (10). The current study determined that up to 68.7% of patients failed to achieve remission at the visit preceding remission solely due to prednisone dose >5 mg/day, while the proportions of patients who did not meet the specific criteria of cSLEDAI-2K=0 or PGA<0.5 were significantly lower, at 5.7% and 8.4%, respectively. Notably, the prednisone dose at the time of LLDAS attainment was not found to be a predictor for subsequent remission. These findings suggest that factors affecting prednisone tapering, rather than the prednisone itself, are the primary determinants of SLE remission. Previous studies have established that patients maintaining long-term hydroxychloroquine use had an increased likelihood of sustaining remission (28, 29). Our data further suggest that continuation of hydroxychloroquine treatment upon LLDAS attainment is associated with accelerated remission. This accelerated remission could potentially be explained by the steroid-sparing effects of hydroxychloroquine.

Lastly, we identified several independent factors influencing remission following the attainment of LLDAS. 1) Age at disease onset. Advanced age at disease onset was associated with a reduced time to remission. 2) Clinical features. Arthritis or gastrointestinal involvement correlated with a shorter time to remission, whereas anemia was associated with a prolonged time to remission. These findings are largely congruent with the results of previous studies in this field.

This study has several inherent limitations related to its retrospective cohort design. First, the lack of standardized follow-up intervals and potential underreporting of changes in disease status between visits may have led to delays in detecting target achievement or flares. These data collection gaps were further exacerbated by the retrospective substitution of missing values with historical results, a methodological constraint that could influence outcome timelines. Second, the absence of a predefined steroid tapering protocol in this real-world setting meant that physicians’ individualized treatment decisions—while reflective of clinical practice—might have introduced variability in time-to-remission assessments. Nevertheless, our findings consistently identified prednisone dosage as the primary determinant of remission outcomes across analyses, suggesting that these limitations likely had minimal impact on the core conclusions regarding steroid dependency.

In conclusion, this study demonstrated that achieving remission after LLDAS attainment remains challenging for the majority of SLE patients. Disease activity at the time of LLDAS attainment significantly influences the time to achieve remission, with the primary barrier to remission being the inability to reduce prednisone daily dose. The study identified several independent factors influencing remission following the attainment of LLDAS, including age at disease onset, clinical manifestations, and maintenance therapy.

## Data Availability

The raw data supporting the conclusions of this article will be made available by the authors, without undue reservation.
