# Supplementary material for: Transitioning from Lupus Low Disease Activity State to remission in systemic lupus erythematosus: real-world evidence
Source: Front Immunol. 2025 Mar 20;16:1546306. doi: 10.3389/fimmu.2025.1546306 (PMC11965674; doi:10.3389/fimmu.2025.1546306)
Supplement: Supplementary file 1 [file Table1.docx]

| **Supplement Table 1** Demographic and clinical characteristics between patients who achieved remission or flare earlier after LLDAS attainment | | | |
| --- | --- | --- | --- |
| **Characteristics** | **Remission before flare**  **n=222** | **Flare before remission**  **n=132** | ***P*-value*** |
| **Female** | 196 (88.3) | 119 (90.2) | 0.726 |
| **Age at disease onset, years** | 31.2 (23.5-45.7) | 28.1 (21.6-34.6) | **0.003** |
| **Age at LLDAS, years** | 38.4 (28.0-51.2) | 33.5 (27.3-41.0) | **0.012** |
| **Disease duration at LLDAS, years** | 3.1 (1.8-7.0) | 4.2 (1.9-9.2) | 0.104 |
| **Comorbidities** |  |  |  |
| SS | 34 (15.3) | 19 (14.4) | 0.878 |
| APS | 9 (4.1) | 7 (5.3) | 0.604 |
| **Clinical manifestations ever** |  |  |  |
| Fever | 43 (19.4) | 31 (23.5) | 0.418 |
| Mucosal ulcers | 41 (18.5) | 31 (23.5) | 0.276 |
| Alopecia | 93 (41.9) | 60 (45.5) | 0.579 |
| Rash | 113 (50.9) | 85 (64.4) | **0.015** |
| Raynaud's phenomenon | 44 (19.8) | 22 (16.7) | 0.484 |
| Arthritis | 51 (23.0) | 24 (18.2) | 0.347 |
| Myositis | 9 (4.1) | 7 (5.3) | 0.604 |
| Serositis | 53 (23.9) | 24 (18.2) | 0.232 |
| Neuropsychiatric manifestations | 26 (11.7) | 16 (12.1) | >0.999 |
| Nephritis | 124 (55.9) | 73 (55.3) | >0.999 |
| Haemolytic anaemia | 8 (3.6) | 13 (9.8) | **0.020** |
| Gastrointestinal involvement | 19 (8.6) | 3 (2.3) | **0.021** |
| **Laboratory abnormal ever** |  |  |  |
| ANA | 219 (98.6) | 132 (100) | 0.296 |
| Anti-Sm | 50 (22.5) | 31 (23.5) | 0.896 |
| Anti-nRNP | 91 (41.0) | 60 (45.5) | 0.438 |
| Anti-rRNP | 62 (27.9) | 40 (30.3) | 0.630 |
| Anti-SSA | 126 (56.8) | 78 (59.1) | 0.739 |
| Anti-SSB | 33 (14.9) | 23 (17.4) | 0.549 |
| Anti-dsDNA | 188 (84.7) | 116 (87.9) | 0.434 |
| Hypocomplementemia | 181 (81.5) | 118 (89.4) | 0.050 |
| Anaemia | 162 (73.0) | 105 (79.5) | 0.202 |
| Leukopenia | 76 (34.2) | 72 (54.5) | **<0.001** |
| Thrombocytopenia | 51 (23.0) | 44 (33.3) | **0.036** |
| Elevated serum creatinine | 17 (7.7) | 14 (10.6) | 0.339 |
| **Disease activity at LLDAS** |  |  |  |
| PGA | 0.3 (0.2-0.4) | 0.4 (0.2-0.5) | **<0.001** |
| SLEDAI-2K** | 2 (0-2) | 2 (1-3) | **<0.001** |
| Alopecia | 3 (1.4) | 2 (1.5) | >0.999 |
| Rash | 1 (0.5) | 6 (4.5) | **0.012** |
| Anti-dsDNA positive | 96 (43.2) | 78 (59.1) | **0.004** |
| Hypocomplementemia | 38 (17.1) | 43 (32.6) | **0.001** |
| Leukopenia | 3 (1.4) | 4 (3.0) | 0.431 |
| Thrombocytopenia | 3 (1.4) | 6 (4.5) | 0.084 |
| **SDI at LLDAS** | 0.5 (0-2) | 0.5 (0-2) | >0.999 |
| **Treatments at LLDAS** |  |  |  |
| Prednisone (or equivalent) dose at LLDAS, mg/day | 7.5 (7.5-7.5) | 7.5 (7.5-7.5) | 0.477 |
| Hydroxychloroquine | 203 (91.4) | 114 (86.4) | 0.152 |
| Immunosuppressants | 155 (69.8) | 86 (65.2) | 0.409 |
| Mycophenolate mofetil | 52 (23.4) | 43 (32.6) | 0.064 |
| Azathioprine | 44 (19.8) | 18 (13.6) | 0.151 |
| Methotrexate | 21 (9.5) | 13 (9.8) | >0.999 |
| Leflunomide | 19 (8.6) | 7 (5.3) | 0.298 |
| Cyclosporine A | 8 (3.6) | 9 (6.8) | 0.202 |
| Cyclophosphamide | 14 (6.3) | 1 (0.8) | **0.012** |
| Tacrolimus | 2 (0.9) | 1 (0.8) | >0.999 |
| * Mann-Whitney U test or Fisher's exact test. **Items of SLEDAI-2K not shown were all negative. Abbreviation: LLDAS: Lupus Low Disease Activity State; SS: Sjogren's syndrome; APS: antiphospholipid syndrome; ANA: antinuclear antibody; PGA: physician’s global assessment; SLEDAI-2K: systemic lupus erythematosus disease activity index 2000; SDI: Systemic Lupus International Collaborating Clinics/American College of Rheumatology Damage Index. | | | |

| **Supplement Table 2** Factors associated with time to remission after LLDAS attainment in 369 patients based on univariable Cox model | | |
| --- | --- | --- |
| **Factors** | **HR (95% CI)** | ***P*-value** |
| **Female** | 0.909 (0.635-1.300) | 0.599 |
| **Age at disease onset, years** | 1.012 (1.004-1.019) | **0.002** |
| **Age at LLDAS, years** | 1.009 (1.001-1.017) | **0.024** |
| **Disease duration at LLDAS, years** | 0.977 (0.956-0.998) | **0.035** |
| **Comorbidities** |  |  |
| Sjogren's syndrome | 1.088 (0.789-1.502) | 0.606 |
| Antiphospholipid syndrome | 0.874 (0.501-1.526) | 0.637 |
| **Clinical manifestations ever** |  |  |
| Fever | 0.885 (0.668-1.174) | 0.397 |
| Mucosal ulcers | 0.923 (0.692-1.230) | 0.583 |
| Alopecia | 0.898 (0.713-1.131) | 0.362 |
| Rash | 0.844 (0.672-1.062) | **0.147** |
| Raynaud's phenomenon | 0.926 (0.692-1.240) | 0.608 |
| Arthritis | 1.278 (0.971-1.681) | **0.080** |
| Myositis | 0.932 (0.534-1.627) | 0.805 |
| Serositis | 1.432 (1.095-1.872) | **0.009** |
| Neuropsychiatric manifestations | 1.284 (0.909-1.814) | **0.156** |
| Nephritis | 1.230 (0.977-1.549) | **0.078** |
| Haemolytic anaemia | 0.821 (0.503-1.341) | 0.432 |
| Gastrointestinal involvement | 2.095 (1.335-3.288) | **0.001** |
| **Laboratory abnormal ever** |  |  |
| ANA | 0.352 (0.112-1.105) | **0.074** |
| Anti-Sm | 1.020 (0.776-1.340) | 0.887 |
| Anti-nRNP | 0.939 (0.745-1.184) | 0.594 |
| Anti-rRNP | 0.920 (0.717-1.181) | 0.514 |
| Anti-SSA | 0.878 (0.698-1.105) | 0.267 |
| Anti-SSB | 0.884 (0.641-1.219) | 0.451 |
| Anti-dsDNA | 0.700 (0.507-0.966) | **0.030** |
| Hypocomplementemia | 0.635 (0.468-0.860) | **0.003** |
| Anaemia | 0.756 (0.584-0.980) | **0.035** |
| Leukopenia | 0.708 (0.560-0.894) | **0.004** |
| Thrombocytopenia | 0.829 (0.639-1.074) | 0.155 |
| Elevated serum creatinine | 1.093 (0.736-1.624) | 0.659 |
| **Disease activity at LLDAS** |  |  |
| PGA | 0.095 (0.048-0.186) | **<0.001** |
| SLEDAI-2K | 0.744 (0.652-0.776) | **<0.001** |
| Alopecia | 0.935 (0.348-2.509) | 0.894 |
| Rash | 0.252 (0.080-0.794) | **0.019** |
| Anti-dsDNA positive | 0.584 (0.464-0.737) | **<0.001** |
| Hypocomplementemia | 0.483 (0.360-0.648) | **<0.001** |
| Leukopenia | 0.697 (0.288-1.688) | 0.424 |
| Thrombocytopenia | 0.244 (0.091-0.656) | **0.005** |
| **SDI at LLDAS** | 1.019 (0.951-1.092) | 0.596 |
| **Treatments at LLDAS** |  |  |
| Prednisone (or equivalent) dose at LLDAS, mg/day | 1.067 (0.939-1.211) | 0.319 |
| Hydroxychloroquine | 1.380 (0.934-2.037) | **0.106** |
| Immunosuppressants | 1.204 (0.945-1.534) | **0.133** |
| Mycophenolate mofetil | 1.010 (0.779-1.310) | 0.941 |
| Azathioprine | 0.999 (0.742-1.345) | 0.993 |
| Methotrexate | 0.935 (0.625-1.400) | 0.744 |
| Leflunomide | 1.539 (0.995-2.383) | **0.053** |
| Cyclosporine A | 0.613 (0.335-1.119) | **0.111** |
| Cyclophosphamide | 3.579 (2.106-6.085) | **<0.001** |
| Tacrolimus | 0.792 (0.197-3.185) | 0.743 |
| Abbreviation: LLDAS: Lupus Low Disease Activity State; SS: Sjogren's syndrome; APS: antiphospholipid syndrome; ANA: antinuclear antibody; PGA: physician’s global assessment; SLEDAI-2K: systemic lupus erythematosus disease activity index 2000; SDI: Systemic Lupus International Collaborating Clinics/American College of Rheumatology Damage Index. | | |
